# Supplementary material for: Modeling glioblastoma heterogeneity as a dynamic network of cell states
Source: Mol Syst Biol. 2021 Sep 16;17(9):e10105. doi: 10.15252/msb.202010105 (PMC8444284; doi:10.15252/msb.202010105)
Supplement: Supplementary file 5 — Source Data for Figure 3 [file MSB-17-e10105-s001.zip › Figure3A_sourcedata/GSEA_3065/nosigdb_state2.GseaPreranked.1622718419215/DOROSZKO_U3180MG_CLUSTER_2.html]

Details for gene set DOROSZKO\_U3180MG\_CLUSTER\_2[GSEA]

|  || Dataset | state2 |
| Phenotype | NoPhenotypeAvailable |
| Upregulated in class | na\_neg |
| GeneSet | DOROSZKO\_U3180MG\_CLUSTER\_2 |
| Enrichment Score (ES) | -0.6424757 |
| Normalized Enrichment Score (NES) | -2.9691744 |
| Nominal p-value | 0.0 |
| FDR q-value | 0.0 |
| FWER p-Value | 0.0 |
Table: GSEA Results Summary

  

Fig 1: Enrichment plot: DOROSZKO\_U3180MG\_CLUSTER\_2      
 Profile of the Running ES Score & Positions of GeneSet Members on the Rank Ordered List

  

| PROBE | GENE SYMBOL | GENE\_TITLE | RANK IN GENE LIST | RANK METRIC SCORE | RUNNING ES | CORE ENRICHMENT || 1 | SRSF7 |  |  | 155 | 0.306 | -0.0113 | No |
| 2 | NASP |  |  | 156 | 0.306 | -0.0053 | No |
| 3 | HSPD1 |  |  | 176 | 0.294 | -0.0017 | No |
| 4 | SRSF2 |  |  | 213 | 0.275 | -0.0004 | No |
| 5 | PGP |  |  | 244 | 0.254 | 0.0012 | No |
| 6 | CEBPZ |  |  | 310 | 0.229 | -0.0016 | No |
| 7 | NOP56 |  |  | 396 | 0.201 | -0.0071 | No |
| 8 | TRA2B |  |  | 515 | 0.178 | -0.0168 | No |
| 9 | RIF1 |  |  | 552 | 0.172 | -0.0175 | No |
| 10 | HMGXB4 |  |  | 567 | 0.169 | -0.0158 | No |
| 11 | BCLAF1 |  |  | 642 | 0.158 | -0.0209 | No |
| 12 | SRSF10 |  |  | 664 | 0.155 | -0.0203 | No |
| 13 | ABCE1 |  |  | 746 | 0.144 | -0.0265 | No |
| 14 | IFRD2 |  |  | 841 | 0.134 | -0.0343 | No |
| 15 | SYNE2 |  |  | 853 | 0.132 | -0.0330 | No |
| 16 | PCM1 |  |  | 861 | 0.131 | -0.0312 | No |
| 17 | PTBP1 |  |  | 943 | 0.124 | -0.0378 | No |
| 18 | TCERG1 |  |  | 951 | 0.123 | -0.0362 | No |
| 19 | SFPQ |  |  | 967 | 0.122 | -0.0355 | No |
| 20 | U2SURP |  |  | 999 | 0.120 | -0.0366 | No |
| 21 | PSRC1 |  |  | 1023 | 0.118 | -0.0369 | No |
| 22 | GNL3 |  |  | 1084 | 0.112 | -0.0414 | No |
| 23 | DNAJC2 |  |  | 1087 | 0.112 | -0.0394 | No |
| 24 | DDX46 |  |  | 1118 | 0.110 | -0.0406 | No |
| 25 | ZNF680 |  |  | 1204 | 0.104 | -0.0480 | No |
| 26 | CHD1 |  |  | 1265 | 0.101 | -0.0528 | No |
| 27 | SRSF1 |  |  | 1315 | 0.097 | -0.0563 | No |
| 28 | USP16 |  |  | 1318 | 0.097 | -0.0547 | No |
| 29 | SMCHD1 |  |  | 1328 | 0.096 | -0.0538 | No |
| 30 | HNRNPU |  |  | 1369 | 0.094 | -0.0564 | No |
| 31 | PRPSAP1 |  |  | 1394 | 0.092 | -0.0573 | No |
| 32 | HSPH1 |  |  | 1399 | 0.092 | -0.0560 | No |
| 33 | ABI2 |  |  | 1402 | 0.092 | -0.0544 | No |
| 34 | WEE1 |  |  | 1617 | 0.081 | -0.0766 | No |
| 35 | DDX42 |  |  | 1632 | 0.080 | -0.0766 | No |
| 36 | ABHD5 |  |  | 1650 | 0.079 | -0.0770 | No |
| 37 | YBX3 |  |  | 1677 | 0.078 | -0.0784 | No |
| 38 | TERF1 |  |  | 1781 | 0.073 | -0.0884 | No |
| 39 | GADD45A |  |  | 1791 | 0.073 | -0.0880 | No |
| 40 | LARS |  |  | 1838 | 0.071 | -0.0918 | No |
| 41 | PSMC6 |  |  | 1874 | 0.070 | -0.0943 | No |
| 42 | FAM120A |  |  | 2030 | 0.063 | -0.1103 | No |
| 43 | KNOP1 |  |  | 2122 | 0.060 | -0.1193 | No |
| 44 | HSPA4 |  |  | 2227 | 0.056 | -0.1298 | No |
| 45 | PRRC2A |  |  | 2238 | 0.056 | -0.1298 | No |
| 46 | MTA1 |  |  | 2245 | 0.055 | -0.1294 | No |
| 47 | NAA15 |  |  | 2324 | 0.053 | -0.1371 | No |
| 48 | EWSR1 |  |  | 2352 | 0.052 | -0.1391 | No |
| 49 | TDG |  |  | 2370 | 0.051 | -0.1400 | No |
| 50 | THAP5 |  |  | 2394 | 0.051 | -0.1415 | No |
| 51 | ANAPC5 |  |  | 2529 | 0.047 | -0.1555 | No |
| 52 | MLF1 |  |  | 2541 | 0.046 | -0.1559 | No |
| 53 | ARIH2 |  |  | 2543 | 0.046 | -0.1551 | No |
| 54 | CCDC82 |  |  | 2564 | 0.046 | -0.1564 | No |
| 55 | COMMD2 |  |  | 2652 | 0.043 | -0.1653 | No |
| 56 | SAFB2 |  |  | 2666 | 0.043 | -0.1659 | No |
| 57 | MAT2A |  |  | 2737 | 0.041 | -0.1729 | No |
| 58 | PBRM1 |  |  | 2776 | 0.040 | -0.1764 | No |
| 59 | IFRD1 |  |  | 2840 | 0.038 | -0.1827 | No |
| 60 | NAV1 |  |  | 2863 | 0.037 | -0.1844 | No |
| 61 | PHF20 |  |  | 2926 | 0.036 | -0.1906 | No |
| 62 | EMD |  |  | 2933 | 0.035 | -0.1906 | No |
| 63 | DNM1L |  |  | 3024 | 0.033 | -0.1999 | No |
| 64 | BDP1 |  |  | 3068 | 0.032 | -0.2041 | No |
| 65 | NIN |  |  | 3079 | 0.032 | -0.2046 | No |
| 66 | DAAM1 |  |  | 3107 | 0.031 | -0.2070 | No |
| 67 | USP33 |  |  | 3194 | 0.030 | -0.2160 | No |
| 68 | SNHG9 |  |  | 3222 | 0.029 | -0.2184 | No |
| 69 | IAH1 |  |  | 3230 | 0.029 | -0.2187 | No |
| 70 | NELFCD |  |  | 3233 | 0.029 | -0.2183 | No |
| 71 | ARHGAP5 |  |  | 3234 | 0.029 | -0.2177 | No |
| 72 | LIMA1 |  |  | 3279 | 0.028 | -0.2221 | No |
| 73 | DHX30 |  |  | 3437 | 0.025 | -0.2391 | No |
| 74 | RAF1 |  |  | 3447 | 0.025 | -0.2396 | No |
| 75 | GPBP1 |  |  | 3487 | 0.024 | -0.2435 | No |
| 76 | USP3 |  |  | 3512 | 0.024 | -0.2457 | No |
| 77 | ANAPC7 |  |  | 3535 | 0.023 | -0.2477 | No |
| 78 | PNN |  |  | 3632 | 0.022 | -0.2580 | No |
| 79 | HNRNPH1 |  |  | 3633 | 0.022 | -0.2576 | No |
| 80 | RBM25 |  |  | 3654 | 0.021 | -0.2594 | No |
| 81 | TLK1 |  |  | 3656 | 0.021 | -0.2591 | No |
| 82 | TIMM44 |  |  | 3736 | 0.020 | -0.2675 | No |
| 83 | RBM28 |  |  | 3786 | 0.019 | -0.2726 | No |
| 84 | TUFM |  |  | 3799 | 0.019 | -0.2736 | No |
| 85 | CWC25 |  |  | 3828 | 0.018 | -0.2763 | No |
| 86 | SRSF11 |  |  | 3837 | 0.018 | -0.2769 | No |
| 87 | HOOK3 |  |  | 3838 | 0.018 | -0.2765 | No |
| 88 | MYC |  |  | 3842 | 0.018 | -0.2765 | No |
| 89 | YME1L1 |  |  | 3852 | 0.018 | -0.2771 | No |
| 90 | SF3B1 |  |  | 3915 | 0.017 | -0.2837 | No |
| 91 | DNPEP |  |  | 3942 | 0.016 | -0.2863 | No |
| 92 | SNRNP70 |  |  | 3992 | 0.016 | -0.2914 | No |
| 93 | ATXN7L3B |  |  | 4009 | 0.015 | -0.2929 | No |
| 94 | CNOT2 |  |  | 4026 | 0.015 | -0.2944 | No |
| 95 | CCDC88A |  |  | 4035 | 0.015 | -0.2950 | No |
| 96 | OSBPL1A |  |  | 4105 | 0.014 | -0.3024 | No |
| 97 | DDX3X |  |  | 4155 | 0.013 | -0.3076 | No |
| 98 | SMARCC2 |  |  | 4179 | 0.012 | -0.3100 | No |
| 99 | LUC7L |  |  | 4191 | 0.012 | -0.3109 | No |
| 100 | RB1CC1 |  |  | 4277 | 0.011 | -0.3202 | No |
| 101 | SREK1 |  |  | 4426 | 0.008 | -0.3365 | No |
| 102 | TBL1XR1 |  |  | 4453 | 0.008 | -0.3393 | No |
| 103 | NCOR1 |  |  | 4520 | 0.007 | -0.3465 | No |
| 104 | SRSF6 |  |  | 4555 | 0.006 | -0.3502 | No |
| 105 | ELMSAN1 |  |  | 4700 | 0.004 | -0.3661 | No |
| 106 | RBM26 |  |  | 4724 | 0.004 | -0.3686 | No |
| 107 | CEBPZOS |  |  | 4877 | 0.001 | -0.3855 | No |
| 108 | TUBGCP2 |  |  | 4895 | 0.001 | -0.3874 | No |
| 109 | AUP1 |  |  | 4931 | 0.001 | -0.3913 | No |
| 110 | RPRD1A |  |  | 4944 | 0.001 | -0.3926 | No |
| 111 | RBM23 |  |  | 4989 | -0.000 | -0.3975 | No |
| 112 | ABHD12 |  |  | 5028 | -0.000 | -0.4017 | No |
| 113 | PJA2 |  |  | 5030 | -0.000 | -0.4018 | No |
| 114 | HSPA1B |  |  | 5070 | -0.001 | -0.4061 | No |
| 115 | CHMP4A |  |  | 5110 | -0.002 | -0.4105 | No |
| 116 | GDAP1 |  |  | 5122 | -0.002 | -0.4116 | No |
| 117 | DDB1 |  |  | 5130 | -0.002 | -0.4124 | No |
| 118 | MED6 |  |  | 5174 | -0.003 | -0.4171 | No |
| 119 | KIF21A |  |  | 5342 | -0.006 | -0.4356 | No |
| 120 | CSNK2A1 |  |  | 5362 | -0.006 | -0.4376 | No |
| 121 | SECISBP2 |  |  | 5398 | -0.007 | -0.4414 | No |
| 122 | PHF14 |  |  | 5454 | -0.008 | -0.4474 | No |
| 123 | CDC37 |  |  | 5466 | -0.008 | -0.4484 | No |
| 124 | AHCYL1 |  |  | 5638 | -0.011 | -0.4673 | No |
| 125 | MTF2 |  |  | 5648 | -0.011 | -0.4681 | No |
| 126 | QTRT1 |  |  | 5653 | -0.011 | -0.4683 | No |
| 127 | ORAI2 |  |  | 5669 | -0.011 | -0.4697 | No |
| 128 | CCDC14 |  |  | 5679 | -0.011 | -0.4705 | No |
| 129 | B3GAT3 |  |  | 5697 | -0.011 | -0.4722 | No |
| 130 | ZNF131 |  |  | 5745 | -0.012 | -0.4772 | No |
| 131 | FOXN2 |  |  | 5769 | -0.013 | -0.4795 | No |
| 132 | FAM172A |  |  | 5800 | -0.013 | -0.4826 | No |
| 133 | DDX18 |  |  | 5826 | -0.014 | -0.4851 | No |
| 134 | MYCBP2 |  |  | 5830 | -0.014 | -0.4852 | No |
| 135 | RGS12 |  |  | 5836 | -0.014 | -0.4855 | No |
| 136 | MAGI1 |  |  | 5887 | -0.014 | -0.4908 | No |
| 137 | PTK2 |  |  | 5931 | -0.015 | -0.4953 | No |
| 138 | PUS7L |  |  | 5957 | -0.016 | -0.4977 | No |
| 139 | REV3L |  |  | 5959 | -0.016 | -0.4975 | No |
| 140 | TMEM120A |  |  | 5982 | -0.016 | -0.4997 | No |
| 141 | ESF1 |  |  | 6012 | -0.017 | -0.5026 | No |
| 142 | HECTD1 |  |  | 6015 | -0.017 | -0.5025 | No |
| 143 | CAND1 |  |  | 6054 | -0.017 | -0.5064 | No |
| 144 | PPP1R16A |  |  | 6146 | -0.019 | -0.5161 | No |
| 145 | SUPT20H |  |  | 6157 | -0.019 | -0.5169 | No |
| 146 | CELF1 |  |  | 6168 | -0.019 | -0.5176 | No |
| 147 | PTCD3 |  |  | 6180 | -0.020 | -0.5185 | No |
| 148 | GNPAT |  |  | 6186 | -0.020 | -0.5186 | No |
| 149 | HES1 |  |  | 6191 | -0.020 | -0.5187 | No |
| 150 | FUS |  |  | 6216 | -0.020 | -0.5210 | No |
| 151 | GPAA1 |  |  | 6264 | -0.021 | -0.5258 | No |
| 152 | CCDC66 |  |  | 6301 | -0.022 | -0.5294 | No |
| 153 | DTX3 |  |  | 6311 | -0.022 | -0.5300 | No |
| 154 | CCS |  |  | 6365 | -0.023 | -0.5354 | No |
| 155 | ZNF800 |  |  | 6367 | -0.023 | -0.5351 | No |
| 156 | PKN2 |  |  | 6401 | -0.024 | -0.5383 | No |
| 157 | CTTN |  |  | 6421 | -0.024 | -0.5400 | No |
| 158 | DDIT3 |  |  | 6433 | -0.024 | -0.5407 | No |
| 159 | TIA1 |  |  | 6487 | -0.025 | -0.5461 | No |
| 160 | ZNF207 |  |  | 6497 | -0.025 | -0.5467 | No |
| 161 | SNRNP200 |  |  | 6581 | -0.027 | -0.5554 | No |
| 162 | PRPF4B |  |  | 6598 | -0.027 | -0.5566 | No |
| 163 | VPS37B |  |  | 6609 | -0.027 | -0.5572 | No |
| 164 | RBM4B |  |  | 6624 | -0.028 | -0.5582 | No |
| 165 | RSBN1L |  |  | 6643 | -0.028 | -0.5597 | No |
| 166 | RAB3IP |  |  | 6660 | -0.029 | -0.5609 | No |
| 167 | ARID4A |  |  | 6708 | -0.030 | -0.5656 | No |
| 168 | KMT2A |  |  | 6717 | -0.030 | -0.5659 | No |
| 169 | KMT5B |  |  | 6746 | -0.030 | -0.5684 | No |
| 170 | RIMKLB |  |  | 6830 | -0.032 | -0.5770 | No |
| 171 | TLE4 |  |  | 6872 | -0.033 | -0.5810 | No |
| 172 | PRPF38B |  |  | 6893 | -0.034 | -0.5825 | No |
| 173 | SLC35F1 |  |  | 6914 | -0.035 | -0.5841 | No |
| 174 | TCF25 |  |  | 6919 | -0.035 | -0.5838 | No |
| 175 | XBP1 |  |  | 6931 | -0.035 | -0.5844 | No |
| 176 | FERMT1 |  |  | 6963 | -0.035 | -0.5872 | No |
| 177 | IST1 |  |  | 6968 | -0.036 | -0.5869 | No |
| 178 | TRMT13 |  |  | 6975 | -0.036 | -0.5869 | No |
| 179 | DDX6 |  |  | 6977 | -0.036 | -0.5863 | No |
| 180 | EPN2 |  |  | 7019 | -0.037 | -0.5901 | No |
| 181 | CLK1 |  |  | 7022 | -0.037 | -0.5896 | No |
| 182 | SLC39A1 |  |  | 7028 | -0.037 | -0.5895 | No |
| 183 | EIF2S3 |  |  | 7040 | -0.038 | -0.5900 | No |
| 184 | DHX36 |  |  | 7071 | -0.038 | -0.5926 | No |
| 185 | UBR5 |  |  | 7104 | -0.039 | -0.5954 | No |
| 186 | KIF1B |  |  | 7160 | -0.041 | -0.6007 | No |
| 187 | MPHOSPH8 |  |  | 7161 | -0.041 | -0.5999 | No |
| 188 | NEMF |  |  | 7163 | -0.041 | -0.5992 | No |
| 189 | TCF4 |  |  | 7166 | -0.041 | -0.5986 | No |
| 190 | FASTK |  |  | 7176 | -0.041 | -0.5988 | No |
| 191 | DMAP1 |  |  | 7195 | -0.042 | -0.6000 | No |
| 192 | CDC42BPA |  |  | 7216 | -0.042 | -0.6014 | No |
| 193 | TMEM87A |  |  | 7231 | -0.043 | -0.6022 | No |
| 194 | RNMT |  |  | 7302 | -0.045 | -0.6091 | No |
| 195 | MTRF1L |  |  | 7314 | -0.045 | -0.6094 | No |
| 196 | DPF3 |  |  | 7344 | -0.046 | -0.6118 | No |
| 197 | DPF2 |  |  | 7347 | -0.046 | -0.6111 | No |
| 198 | PODXL2 |  |  | 7348 | -0.046 | -0.6102 | No |
| 199 | CRTC3 |  |  | 7354 | -0.046 | -0.6099 | No |
| 200 | HAGHL |  |  | 7389 | -0.047 | -0.6127 | No |
| 201 | ATRX |  |  | 7408 | -0.047 | -0.6138 | No |
| 202 | LFNG |  |  | 7443 | -0.048 | -0.6167 | No |
| 203 | RNF115 |  |  | 7464 | -0.049 | -0.6179 | No |
| 204 | TTC37 |  |  | 7469 | -0.050 | -0.6174 | No |
| 205 | TAOK1 |  |  | 7489 | -0.050 | -0.6186 | No |
| 206 | NCK1 |  |  | 7576 | -0.053 | -0.6271 | No |
| 207 | CTNNB1 |  |  | 7580 | -0.053 | -0.6264 | No |
| 208 | ARHGEF7 |  |  | 7591 | -0.053 | -0.6265 | No |
| 209 | MKLN1 |  |  | 7609 | -0.054 | -0.6274 | No |
| 210 | GPATCH2 |  |  | 7627 | -0.054 | -0.6282 | No |
| 211 | ACADVL |  |  | 7628 | -0.054 | -0.6271 | No |
| 212 | F3 |  |  | 7662 | -0.055 | -0.6298 | No |
| 213 | TAOK3 |  |  | 7664 | -0.055 | -0.6288 | No |
| 214 | ARFGAP2 |  |  | 7686 | -0.056 | -0.6300 | No |
| 215 | STX16 |  |  | 7727 | -0.057 | -0.6334 | No |
| 216 | ZNF397 |  |  | 7735 | -0.057 | -0.6331 | No |
| 217 | SRSF5 |  |  | 7743 | -0.058 | -0.6327 | No |
| 218 | TRIM44 |  |  | 7782 | -0.059 | -0.6358 | No |
| 219 | WDR60 |  |  | 7789 | -0.059 | -0.6353 | No |
| 220 | CNTN1 |  |  | 7792 | -0.059 | -0.6344 | No |
| 221 | COLGALT2 |  |  | 7803 | -0.060 | -0.6343 | No |
| 222 | NSUN5 |  |  | 7812 | -0.060 | -0.6340 | No |
| 223 | NPAS3 |  |  | 7814 | -0.060 | -0.6330 | No |
| 224 | FAM53C |  |  | 7829 | -0.060 | -0.6334 | No |
| 225 | THOC2 |  |  | 7856 | -0.061 | -0.6351 | No |
| 226 | USP34 |  |  | 7874 | -0.062 | -0.6358 | No |
| 227 | ZRANB2 |  |  | 7876 | -0.062 | -0.6347 | No |
| 228 | PLPP3 |  |  | 7911 | -0.063 | -0.6372 | No |
| 229 | TNFRSF12A |  |  | 7931 | -0.064 | -0.6381 | No |
| 230 | IFNGR1 |  |  | 7945 | -0.065 | -0.6383 | No |
| 231 | ANK2 |  |  | 7946 | -0.065 | -0.6370 | No |
| 232 | ADAR |  |  | 7980 | -0.066 | -0.6394 | No |
| 233 | ZNF37A |  |  | 7993 | -0.067 | -0.6394 | No |
| 234 | GTF2H2 |  |  | 8002 | -0.067 | -0.6390 | No |
| 235 | ARGLU1 |  |  | 8034 | -0.068 | -0.6411 | Yes |
| 236 | JPX |  |  | 8043 | -0.069 | -0.6407 | Yes |
| 237 | SETD5 |  |  | 8045 | -0.069 | -0.6395 | Yes |
| 238 | CDK5RAP3 |  |  | 8063 | -0.070 | -0.6400 | Yes |
| 239 | SDC3 |  |  | 8064 | -0.070 | -0.6387 | Yes |
| 240 | SPSB3 |  |  | 8082 | -0.070 | -0.6392 | Yes |
| 241 | MATN2 |  |  | 8102 | -0.071 | -0.6399 | Yes |
| 242 | JMJD1C |  |  | 8106 | -0.072 | -0.6388 | Yes |
| 243 | ARHGEF2 |  |  | 8108 | -0.072 | -0.6376 | Yes |
| 244 | ASH1L |  |  | 8113 | -0.072 | -0.6366 | Yes |
| 245 | NOC2L |  |  | 8121 | -0.072 | -0.6360 | Yes |
| 246 | LONP2 |  |  | 8123 | -0.073 | -0.6347 | Yes |
| 247 | GCC2 |  |  | 8126 | -0.073 | -0.6335 | Yes |
| 248 | MARK3 |  |  | 8135 | -0.073 | -0.6329 | Yes |
| 249 | SACS |  |  | 8148 | -0.074 | -0.6328 | Yes |
| 250 | M6PR |  |  | 8183 | -0.076 | -0.6351 | Yes |
| 251 | MCL1 |  |  | 8200 | -0.077 | -0.6354 | Yes |
| 252 | KMT2E |  |  | 8210 | -0.078 | -0.6349 | Yes |
| 253 | RFX4 |  |  | 8211 | -0.078 | -0.6334 | Yes |
| 254 | GALR1 |  |  | 8229 | -0.078 | -0.6337 | Yes |
| 255 | DCLK2 |  |  | 8232 | -0.079 | -0.6324 | Yes |
| 256 | SPPL2A |  |  | 8249 | -0.080 | -0.6327 | Yes |
| 257 | ZNF638 |  |  | 8252 | -0.080 | -0.6313 | Yes |
| 258 | KMT2C |  |  | 8273 | -0.081 | -0.6320 | Yes |
| 259 | TAF1D |  |  | 8275 | -0.081 | -0.6305 | Yes |
| 260 | CREB5 |  |  | 8279 | -0.082 | -0.6293 | Yes |
| 261 | HM13 |  |  | 8294 | -0.083 | -0.6292 | Yes |
| 262 | EXOC4 |  |  | 8300 | -0.083 | -0.6281 | Yes |
| 263 | AFF4 |  |  | 8301 | -0.083 | -0.6265 | Yes |
| 264 | TTC19 |  |  | 8303 | -0.083 | -0.6250 | Yes |
| 265 | SFXN5 |  |  | 8309 | -0.083 | -0.6240 | Yes |
| 266 | ACAP3 |  |  | 8320 | -0.084 | -0.6234 | Yes |
| 267 | GATAD1 |  |  | 8323 | -0.084 | -0.6220 | Yes |
| 268 | SPAG9 |  |  | 8330 | -0.084 | -0.6211 | Yes |
| 269 | PBXIP1 |  |  | 8337 | -0.085 | -0.6201 | Yes |
| 270 | SPECC1 |  |  | 8346 | -0.085 | -0.6193 | Yes |
| 271 | SON |  |  | 8347 | -0.085 | -0.6176 | Yes |
| 272 | NCSTN |  |  | 8349 | -0.085 | -0.6161 | Yes |
| 273 | HEPACAM |  |  | 8380 | -0.088 | -0.6177 | Yes |
| 274 | NSD1 |  |  | 8395 | -0.089 | -0.6176 | Yes |
| 275 | FAM133B |  |  | 8408 | -0.090 | -0.6172 | Yes |
| 276 | GON4L |  |  | 8423 | -0.091 | -0.6170 | Yes |
| 277 | CNOT4 |  |  | 8428 | -0.091 | -0.6156 | Yes |
| 278 | CCNL1 |  |  | 8435 | -0.091 | -0.6145 | Yes |
| 279 | GUSB |  |  | 8446 | -0.092 | -0.6138 | Yes |
| 280 | DDX5 |  |  | 8454 | -0.093 | -0.6128 | Yes |
| 281 | ARRDC3 |  |  | 8461 | -0.093 | -0.6117 | Yes |
| 282 | RBM39 |  |  | 8469 | -0.093 | -0.6106 | Yes |
| 283 | CFLAR |  |  | 8485 | -0.095 | -0.6105 | Yes |
| 284 | SLC25A36 |  |  | 8496 | -0.095 | -0.6097 | Yes |
| 285 | SAR1B |  |  | 8504 | -0.096 | -0.6086 | Yes |
| 286 | TMUB2 |  |  | 8506 | -0.096 | -0.6069 | Yes |
| 287 | TMEM59L |  |  | 8529 | -0.099 | -0.6074 | Yes |
| 288 | RPAP2 |  |  | 8536 | -0.099 | -0.6061 | Yes |
| 289 | SPTLC1 |  |  | 8543 | -0.100 | -0.6049 | Yes |
| 290 | RNF170 |  |  | 8544 | -0.100 | -0.6029 | Yes |
| 291 | MYO6 |  |  | 8550 | -0.100 | -0.6016 | Yes |
| 292 | ZNF292 |  |  | 8563 | -0.101 | -0.6009 | Yes |
| 293 | MFGE8 |  |  | 8573 | -0.101 | -0.6000 | Yes |
| 294 | COPA |  |  | 8599 | -0.103 | -0.6007 | Yes |
| 295 | CAMK2D |  |  | 8602 | -0.103 | -0.5989 | Yes |
| 296 | CPEB4 |  |  | 8604 | -0.104 | -0.5970 | Yes |
| 297 | GPATCH8 |  |  | 8606 | -0.104 | -0.5951 | Yes |
| 298 | AC020916.1 |  |  | 8608 | -0.104 | -0.5932 | Yes |
| 299 | FOXO3 |  |  | 8616 | -0.104 | -0.5920 | Yes |
| 300 | SLC12A9 |  |  | 8618 | -0.104 | -0.5900 | Yes |
| 301 | B4GAT1 |  |  | 8619 | -0.104 | -0.5880 | Yes |
| 302 | SLC25A37 |  |  | 8632 | -0.105 | -0.5873 | Yes |
| 303 | LMNA |  |  | 8636 | -0.106 | -0.5856 | Yes |
| 304 | CTSL |  |  | 8637 | -0.106 | -0.5835 | Yes |
| 305 | DLL3 |  |  | 8640 | -0.106 | -0.5817 | Yes |
| 306 | UNC50 |  |  | 8641 | -0.106 | -0.5796 | Yes |
| 307 | ST5 |  |  | 8647 | -0.107 | -0.5781 | Yes |
| 308 | NCAM1 |  |  | 8660 | -0.108 | -0.5773 | Yes |
| 309 | MSI2 |  |  | 8667 | -0.108 | -0.5759 | Yes |
| 310 | ZNF302 |  |  | 8678 | -0.110 | -0.5749 | Yes |
| 311 | LRP10 |  |  | 8697 | -0.111 | -0.5747 | Yes |
| 312 | MPZL1 |  |  | 8701 | -0.111 | -0.5729 | Yes |
| 313 | IRS2 |  |  | 8704 | -0.111 | -0.5710 | Yes |
| 314 | FNIP1 |  |  | 8716 | -0.112 | -0.5700 | Yes |
| 315 | HERPUD1 |  |  | 8723 | -0.114 | -0.5685 | Yes |
| 316 | PRRC2B |  |  | 8734 | -0.115 | -0.5673 | Yes |
| 317 | OFD1 |  |  | 8748 | -0.117 | -0.5665 | Yes |
| 318 | TRA2A |  |  | 8773 | -0.119 | -0.5669 | Yes |
| 319 | CYHR1 |  |  | 8776 | -0.119 | -0.5648 | Yes |
| 320 | SUMF2 |  |  | 8780 | -0.120 | -0.5628 | Yes |
| 321 | ZEB2 |  |  | 8786 | -0.120 | -0.5610 | Yes |
| 322 | NTM |  |  | 8787 | -0.120 | -0.5587 | Yes |
| 323 | CTSF |  |  | 8795 | -0.122 | -0.5571 | Yes |
| 324 | PCMT1 |  |  | 8796 | -0.122 | -0.5547 | Yes |
| 325 | LUC7L3 |  |  | 8798 | -0.122 | -0.5524 | Yes |
| 326 | TMEM30A |  |  | 8814 | -0.124 | -0.5517 | Yes |
| 327 | ATP6V0B |  |  | 8815 | -0.124 | -0.5493 | Yes |
| 328 | ZNF655 |  |  | 8817 | -0.125 | -0.5470 | Yes |
| 329 | PABPN1 |  |  | 8818 | -0.125 | -0.5445 | Yes |
| 330 | DTNA |  |  | 8828 | -0.126 | -0.5431 | Yes |
| 331 | ASPH |  |  | 8843 | -0.128 | -0.5421 | Yes |
| 332 | CHD6 |  |  | 8845 | -0.128 | -0.5398 | Yes |
| 333 | CD164 |  |  | 8864 | -0.132 | -0.5392 | Yes |
| 334 | GTF2I |  |  | 8867 | -0.132 | -0.5369 | Yes |
| 335 | SAT2 |  |  | 8870 | -0.133 | -0.5345 | Yes |
| 336 | IDS |  |  | 8874 | -0.133 | -0.5322 | Yes |
| 337 | BICD1 |  |  | 8882 | -0.133 | -0.5304 | Yes |
| 338 | MUM1 |  |  | 8888 | -0.134 | -0.5284 | Yes |
| 339 | DDRGK1 |  |  | 8890 | -0.134 | -0.5259 | Yes |
| 340 | MEIS2 |  |  | 8893 | -0.134 | -0.5235 | Yes |
| 341 | PDIA4 |  |  | 8894 | -0.134 | -0.5209 | Yes |
| 342 | CYR61 |  |  | 8901 | -0.136 | -0.5189 | Yes |
| 343 | EXOC7 |  |  | 8905 | -0.137 | -0.5166 | Yes |
| 344 | SF1 |  |  | 8915 | -0.137 | -0.5149 | Yes |
| 345 | SPTAN1 |  |  | 8923 | -0.138 | -0.5130 | Yes |
| 346 | RCN1 |  |  | 8929 | -0.138 | -0.5109 | Yes |
| 347 | EPM2AIP1 |  |  | 8930 | -0.139 | -0.5082 | Yes |
| 348 | ANKRD12 |  |  | 8935 | -0.139 | -0.5059 | Yes |
| 349 | RIC3 |  |  | 8937 | -0.139 | -0.5033 | Yes |
| 350 | SLC22A17 |  |  | 8938 | -0.140 | -0.5006 | Yes |
| 351 | ATP2A2 |  |  | 8946 | -0.141 | -0.4987 | Yes |
| 352 | ARHGAP21 |  |  | 8949 | -0.141 | -0.4961 | Yes |
| 353 | PRKCSH |  |  | 8950 | -0.142 | -0.4934 | Yes |
| 354 | GPRC5B |  |  | 8956 | -0.142 | -0.4912 | Yes |
| 355 | BAZ2B |  |  | 8963 | -0.143 | -0.4890 | Yes |
| 356 | CTSA |  |  | 8964 | -0.143 | -0.4863 | Yes |
| 357 | TM9SF2 |  |  | 8969 | -0.145 | -0.4839 | Yes |
| 358 | SLC16A1 |  |  | 8970 | -0.145 | -0.4811 | Yes |
| 359 | PPP1CB |  |  | 8977 | -0.145 | -0.4789 | Yes |
| 360 | CCDC47 |  |  | 8988 | -0.147 | -0.4772 | Yes |
| 361 | PPIB |  |  | 8991 | -0.147 | -0.4745 | Yes |
| 362 | JAG1 |  |  | 8993 | -0.148 | -0.4718 | Yes |
| 363 | OS9 |  |  | 9006 | -0.150 | -0.4702 | Yes |
| 364 | WDR73 |  |  | 9008 | -0.150 | -0.4674 | Yes |
| 365 | TNRC6B |  |  | 9022 | -0.152 | -0.4659 | Yes |
| 366 | RAB6A |  |  | 9032 | -0.154 | -0.4638 | Yes |
| 367 | POR |  |  | 9033 | -0.155 | -0.4608 | Yes |
| 368 | FGFR1 |  |  | 9034 | -0.155 | -0.4578 | Yes |
| 369 | SLC1A3 |  |  | 9052 | -0.158 | -0.4566 | Yes |
| 370 | SERPINH1 |  |  | 9054 | -0.159 | -0.4537 | Yes |
| 371 | SPG7 |  |  | 9055 | -0.159 | -0.4506 | Yes |
| 372 | EFHC1 |  |  | 9059 | -0.159 | -0.4478 | Yes |
| 373 | CHD9 |  |  | 9066 | -0.160 | -0.4453 | Yes |
| 374 | PRKCA |  |  | 9072 | -0.161 | -0.4428 | Yes |
| 375 | RTN4 |  |  | 9099 | -0.166 | -0.4424 | Yes |
| 376 | RDH11 |  |  | 9108 | -0.167 | -0.4401 | Yes |
| 377 | SPTBN1 |  |  | 9111 | -0.169 | -0.4370 | Yes |
| 378 | AIG1 |  |  | 9113 | -0.169 | -0.4338 | Yes |
| 379 | PNISR |  |  | 9119 | -0.170 | -0.4311 | Yes |
| 380 | SELENOS |  |  | 9135 | -0.174 | -0.4293 | Yes |
| 381 | IP6K2 |  |  | 9136 | -0.174 | -0.4259 | Yes |
| 382 | TMEM165 |  |  | 9138 | -0.175 | -0.4226 | Yes |
| 383 | LRPAP1 |  |  | 9144 | -0.176 | -0.4198 | Yes |
| 384 | PRCP |  |  | 9153 | -0.178 | -0.4172 | Yes |
| 385 | P4HTM |  |  | 9157 | -0.179 | -0.4141 | Yes |
| 386 | FSTL1 |  |  | 9158 | -0.179 | -0.4106 | Yes |
| 387 | ATP6AP1 |  |  | 9160 | -0.179 | -0.4072 | Yes |
| 388 | PDIA6 |  |  | 9165 | -0.180 | -0.4041 | Yes |
| 389 | HEPN1 |  |  | 9169 | -0.181 | -0.4009 | Yes |
| 390 | ACSL3 |  |  | 9175 | -0.184 | -0.3979 | Yes |
| 391 | DPP6 |  |  | 9181 | -0.185 | -0.3949 | Yes |
| 392 | NPDC1 |  |  | 9188 | -0.186 | -0.3919 | Yes |
| 393 | GOLIM4 |  |  | 9189 | -0.187 | -0.3883 | Yes |
| 394 | SYT11 |  |  | 9197 | -0.189 | -0.3854 | Yes |
| 395 | TTC14 |  |  | 9198 | -0.190 | -0.3817 | Yes |
| 396 | MFSD10 |  |  | 9199 | -0.191 | -0.3780 | Yes |
| 397 | DDOST |  |  | 9213 | -0.198 | -0.3756 | Yes |
| 398 | FKBP10 |  |  | 9215 | -0.199 | -0.3718 | Yes |
| 399 | LMAN1 |  |  | 9216 | -0.199 | -0.3679 | Yes |
| 400 | KTN1 |  |  | 9217 | -0.200 | -0.3641 | Yes |
| 401 | DYNC1H1 |  |  | 9231 | -0.205 | -0.3615 | Yes |
| 402 | ATF6B |  |  | 9233 | -0.205 | -0.3576 | Yes |
| 403 | CLSTN1 |  |  | 9246 | -0.210 | -0.3549 | Yes |
| 404 | PTX3 |  |  | 9249 | -0.212 | -0.3510 | Yes |
| 405 | GRN |  |  | 9251 | -0.214 | -0.3469 | Yes |
| 406 | CDH2 |  |  | 9253 | -0.215 | -0.3429 | Yes |
| 407 | MACF1 |  |  | 9254 | -0.215 | -0.3387 | Yes |
| 408 | NKTR |  |  | 9259 | -0.216 | -0.3349 | Yes |
| 409 | SEC61A1 |  |  | 9261 | -0.217 | -0.3308 | Yes |
| 410 | SLC3A2 |  |  | 9268 | -0.220 | -0.3272 | Yes |
| 411 | GOLGB1 |  |  | 9274 | -0.221 | -0.3234 | Yes |
| 412 | ADGRG1 |  |  | 9275 | -0.222 | -0.3191 | Yes |
| 413 | N4BP2L2 |  |  | 9277 | -0.223 | -0.3149 | Yes |
| 414 | SAT1 |  |  | 9279 | -0.224 | -0.3106 | Yes |
| 415 | FJX1 |  |  | 9282 | -0.225 | -0.3065 | Yes |
| 416 | ADAM9 |  |  | 9285 | -0.226 | -0.3023 | Yes |
| 417 | RRBP1 |  |  | 9287 | -0.227 | -0.2980 | Yes |
| 418 | DDX17 |  |  | 9288 | -0.228 | -0.2936 | Yes |
| 419 | PDIA3 |  |  | 9297 | -0.230 | -0.2900 | Yes |
| 420 | SERINC1 |  |  | 9303 | -0.233 | -0.2860 | Yes |
| 421 | CALR |  |  | 9310 | -0.237 | -0.2821 | Yes |
| 422 | SEMA5A |  |  | 9315 | -0.243 | -0.2778 | Yes |
| 423 | LAMP1 |  |  | 9317 | -0.244 | -0.2732 | Yes |
| 424 | AKAP9 |  |  | 9318 | -0.244 | -0.2684 | Yes |
| 425 | CTSD |  |  | 9320 | -0.246 | -0.2637 | Yes |
| 426 | CD9 |  |  | 9324 | -0.248 | -0.2592 | Yes |
| 427 | STAT3 |  |  | 9325 | -0.249 | -0.2544 | Yes |
| 428 | BSG |  |  | 9331 | -0.253 | -0.2500 | Yes |
| 429 | RCN2 |  |  | 9332 | -0.255 | -0.2451 | Yes |
| 430 | HSP90B1 |  |  | 9334 | -0.257 | -0.2402 | Yes |
| 431 | KANSL1 |  |  | 9335 | -0.259 | -0.2351 | Yes |
| 432 | LRP1 |  |  | 9338 | -0.259 | -0.2303 | Yes |
| 433 | ZBTB20 |  |  | 9341 | -0.261 | -0.2254 | Yes |
| 434 | DDR1 |  |  | 9345 | -0.266 | -0.2206 | Yes |
| 435 | ITGAV |  |  | 9346 | -0.269 | -0.2154 | Yes |
| 436 | NOVA1 |  |  | 9348 | -0.272 | -0.2102 | Yes |
| 437 | PTPRZ1 |  |  | 9351 | -0.272 | -0.2051 | Yes |
| 438 | RSRP1 |  |  | 9355 | -0.278 | -0.2000 | Yes |
| 439 | SPARCL1 |  |  | 9360 | -0.288 | -0.1949 | Yes |
| 440 | ITGB8 |  |  | 9362 | -0.294 | -0.1893 | Yes |
| 441 | PSAP |  |  | 9363 | -0.294 | -0.1836 | Yes |
| 442 | ATP6AP2 |  |  | 9365 | -0.294 | -0.1779 | Yes |
| 443 | LAPTM4A |  |  | 9368 | -0.299 | -0.1723 | Yes |
| 444 | GLG1 |  |  | 9371 | -0.306 | -0.1666 | Yes |
| 445 | CANX |  |  | 9374 | -0.310 | -0.1608 | Yes |
| 446 | LAMP2 |  |  | 9380 | -0.322 | -0.1551 | Yes |
| 447 | GPM6A |  |  | 9381 | -0.324 | -0.1487 | Yes |
| 448 | B2M |  |  | 9383 | -0.331 | -0.1424 | Yes |
| 449 | ITGB1 |  |  | 9388 | -0.339 | -0.1363 | Yes |
| 450 | CALD1 |  |  | 9391 | -0.348 | -0.1297 | Yes |
| 451 | WSB1 |  |  | 9393 | -0.350 | -0.1230 | Yes |
| 452 | VCAN |  |  | 9396 | -0.364 | -0.1162 | Yes |
| 453 | SNTG1 |  |  | 9401 | -0.376 | -0.1093 | Yes |
| 454 | TNC |  |  | 9403 | -0.383 | -0.1019 | Yes |
| 455 | HSPA5 |  |  | 9406 | -0.395 | -0.0945 | Yes |
| 456 | APLP2 |  |  | 9409 | -0.408 | -0.0867 | Yes |
| 457 | COL6A1 |  |  | 9410 | -0.410 | -0.0788 | Yes |
| 458 | APP |  |  | 9412 | -0.426 | -0.0706 | Yes |
| 459 | KCNQ1OT1 |  |  | 9414 | -0.434 | -0.0623 | Yes |
| 460 | VMP1 |  |  | 9417 | -0.448 | -0.0538 | Yes |
| 461 | MEG3 |  |  | 9419 | -0.461 | -0.0449 | Yes |
| 462 | DST |  |  | 9422 | -0.485 | -0.0357 | Yes |
| 463 | IGFBP3 |  |  | 9424 | -0.502 | -0.0260 | Yes |
| 464 | CLU |  |  | 9430 | -0.547 | -0.0159 | Yes |
| 465 | NEAT1 |  |  | 9439 | -0.871 | 0.0001 | Yes |
Table: GSEA details [plain text format]

  

Fig 2: DOROSZKO\_U3180MG\_CLUSTER\_2: Random ES distribution      
 Gene set null distribution of ES for **DOROSZKO\_U3180MG\_CLUSTER\_2**
